# Intra-arterial nimodipine for severe cerebral vasospasm after aneurysmal subarachnoid haemorrhage – neurological and radiological outcome

**DOI:** 10.1177/19714009211036695

**Published:** 2021-08-05

**Authors:** Jennifer Samuelsson, Merete Sunila, Alexandros Rentzos, Daniel Nilsson

**Affiliations:** 1 Institute of Neurosciences and Physiology, Gothenburg University, Sweden; 2 Department of Neurosurgery, Sahlgrenska University Hospital, Sweden; 3 Department of Diagnostic and Interventional Neuroradiology, Sahlgrenska University Hospital, Sweden; 4 Department of Radiology, Institute of Clinical Sciences, University of Gothenburg, Gothenburg, Sweden

**Keywords:** Cerebral vasospasm, intra-arterial nimodipine, subarachnoid haemorrhage, cerebral infarction, complication

## Abstract

**Objectives:**

Cerebral vasospasm is a known complication to aneurysmal subarachnoid haemorrhage, which can lead to severe morbidity. Intra-arterial vasodilation therapy is widely used as a last resort treatment in patients with symptomatic refractory cerebral vasospasm but there is limited data about the outcome. The purpose of this study is to evaluate the neurological and radiological outcome in patients treated with intra-arterial nimodipine in relation to cerebral infarction, procedure-related complications and clinical outcome.

**Methods:**

Patients with refractory cerebral vasospasm treated with intra-arterial nimodipine during 2009–2020 at Sahlgrenska University Hospital were retrospectively reviewed. Neurological outcome (modified Rankin Scale) at 30 days and 6 months, development of cerebral infarction after intra-arterial nimodipine treatment and procedure-related complications were studied.

**Results:**

Forty-eight patients were treated with intra-arterial nimodipine. A good outcome (modified Rankin Scale 0–2) was seen in 25% (*n* = 12) of the patients after 30 days and in 47% (*n* = 22) of the patients after six months. Infarction related to the vasospastic vessel after treatment with intra-arterial nimodipine was seen in 60% (*n* = 29) of the patients. A total of 124 procedures with intra-arterial nimodipine were performed where complications were seen in 10 (21%) patients in 10 (8%) procedures. Four (8%) patients died within 30 days.

**Conclusions:**

A majority of patients developed an ischaemic cerebral infarction in spite of intra-arterial nimodipine treatment. However, a good clinical recovery was seen in almost half of the patients after 6 months. Minor complications occurred in one out of five patients.

## Introduction

Cerebral vasospasm (CVS) occurs in up to 30% of patients after subarachnoid haemorrhage (SAH) and can result in delayed cerebral ischaemia (DCI), brain infarction and death.^1^

DCI occurs in about 30% of patients with CVS, typically between day 4–14 post ictus.^1^ DCI is defined as clinical or radiographic signs of ischaemia and is an important cause of morbidity contributing to poor outcome after SAH when associated with cerebral infarction.^2^ One main cause for DCI has long been considered to be vasospasm identifiable on angiography. Angiographic vasospasm occurs in up to 70% of patients indicating that the relationship between angiography and clinical symptoms can be inconsistent.^3–6^ A known risk factor to develop vasospasm is the extent and size of the initial bleeding, often graded on Fisher scale.^7^

First, smooth muscle contraction in the cerebral arteries causes narrowing of the vessels. Subarachnoid blood triggers calcium flows and downward cellular cascades resulting in smooth muscle contraction through myofilament activation. Second, endothelial factors, such as a decrease of vasodilating factor nitric oxide (NO) or increase of a vasoconstrictor peptide endothelin and imbalance between them, may play an important role. Finally, inflammatory processes after SAH contributing to vasospasm by vasoconstriction have also been studied showing that, besides nimodipine, a reduction in vasospasm-related morbidity and mortality in the past two decades has been due to appropriate fluid management, induced hypertension when indicated for symptomatic ischaemia and balloon angioplasty when deemed necessary.^8^

First-line therapy for vasospasm focuses on improving cerebral blood flow and brain oxygen delivery through induced hypertension and nimodipine administration.^9^ Randomised controlled trials have shown nimodipine to reduce poor neurological outcomes and decrease mortality which is why nimodipine is recommended to patients either orally or intravenously during the first 21 days.^10^ Despite its neuroprotective and vasodilatory benefits, some patients deteriorate further and develop severe cerebral vasospasm.^1^ Medical strategies like the triple-H therapy with haemodilution, hypervolaemia and hypertension has traditionally been used in prevention of vasospasm by raising the mean arterial pressure and increasing cerebral perfusion.^11^ However, studies have failed to show benefit with hypervolaemia compared to euvolaemia and instead increased risk of pulmonary oedema, myocardial ischaemia and cerebral oedema have been described. For this reason, maintenance of euvolaemia and induced hypertension are recommended today.^12^

In cases where patients do not respond to primary treatment and are showing signs of either neurological deficits or radiological vasospasm for those being sedated, rescue therapy with endovascular strategies is considered. Balloon angioplasty has been found effective with long-lasting results, but the procedure is limited to proximal vessels and is associated with risk of vessel rupture and thromboembolic events. Furthermore, the procedure demands an experienced neurointerventionist.^13^ Intra-arterial vasodilators can in turn also be used in more distal and diffuse vasospasms, but these tend to have a temporary effect and require repeated treatments.^5^ Potential side effects such as arterial hypotension and rising ICP Intracranial pressure should also be taken into consideration.

Biondi et al. (2004) demonstrated in the early 2000s that intra-arterial nimodipine (IAN) is an effective treatment for symptomatic vasospasm showing an angiographic dilatation in 43% of patients and improved clinical outcome in 76% of the patients. A few studies later described similar results with short-term angiographic results and clinical improvement after IAN treatment.^1,13–15,16^ Although several studies support the efficacy of endovascular techniques with angiographic and clinical improvement, only limited data exist about repeated treatments and long-term efficacy.

## Materials and methods

### Population

Data was collected retrospectively of patients treated with IAN for symptomatic CVS after SAH at Sahlgrenska University (SU) Hospital during 2009–2020. A register of patients treated with IAN from the Department of Interventional Neuroradiology at SU Hospital was obtained. Forty-eight patients were identified of whom five patients were treated with both IAN and percutaneous transluminal angioplasty (PTA).

Demographic data (age, sex, hypertension, cigarette smoking), clinical condition at admission (WFNS), the severity of SAH (Fisher scale), aneurysm location and treatment were collected from the patient medical records. Neurological status was identified at admission, before IAN and at discharge. Onset and location of CVS on TCD (>200 cm/s) and on a computed tomographic angiography (CTA) evaluated by a neuroradiologist, affected vessels, dosage of IAN, number of repeated procedures and complications related to the procedure were recorded. The presence and localization of infarction or ischaemia and presence of hydrocephalus were identified by an experienced neuroradiologist through computed tomography (CT) or magnetic resonance imaging (MRI) reports.

Clinical outcome was evaluated according to the modified Rankin Scale (mRS) after 30 days and 6 months. The six-month follow-up was chosen due to the patients usually still being hospitalised, defining mRS 0–2 as a good outcome and mRS 3–6 as poor.

The decision to treat the patient with IAN was made by the attending neurosurgeon and the interventional neuroradiologist based on clinical and radiological data. Patients were monitored with TCD Transcranial doppler and in case of elevated velocities and/or clinical signs of vasospasm, both a CT and CTA were conducted. Computed tomography perfusion (CTP) has been added in recent years to detect hypoperfusion and to support indication for treatment with IAN. Only patients with clinical and/or radiological progress of vasospasm despite intravenous nimodipine in an adequate dose according to weight, mean arterial pressure (MAP) of >80 and euvolaemia were considered as candidates for IAN.

Endovascular treatment was performed with the patient under anaesthesia and radiological equipment intended for cerebral angiographies was used. Depending on the CTA result and clinical signs, the catheter was placed in the respective vessel during imaging; usually MCA Middle cerebral artery (first segment), ACA Anterior Cerebral artery (first segment), basilary artery and internal carotid artery (ICA) if both ACA and MCA territories were affected ipsilaterally. The catheter position was controlled with contrast injection.

Nimodipine was injected at 4 mg per treatment session, and sometimes up to 6 mg (in more severe cases up to 8–10 mg per treatment session). Blood pressure was monitored to avoid hypotension.

**Figure 1.**
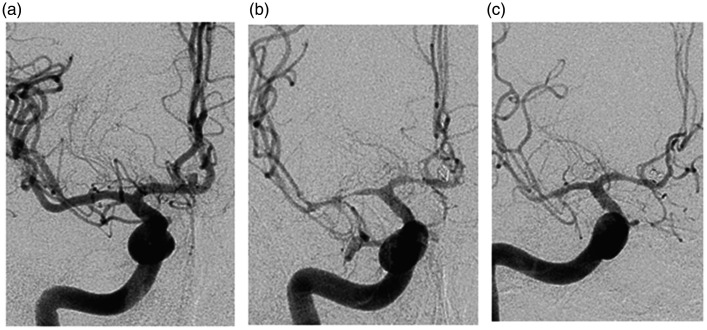
Angiographic examination via the internal carotid artery (ICA) in a patient with subarachnoid haemorrhage (SAH). (a) Angiogram at time of arrival showing an anterior communicant artery aneurysm (ACoA). (b) Severe vasospasm shown on angiogram 4 days post-treatment before intra-arterial nimodipine (IAN). (c) Angiogram immediately after administration of IAN.

If an effect of IAN was seen, the introducer at the puncture site of femoral artery would be left for eventual angiography and treatment either later on the same day or in the upcoming days after (Figure 1). If no effect was seen, the case would be discussed with the attending neurosurgeon to decide if the treatment would be repeated on the same day or if a more aggressive method with PTA should be performed. PTA was performed only if no effect of IAN was seen and patient had a severe vasospasm with symptoms and/or CTP with signs of hypoperfusion.

### Statistical methods

Statistical analysis was performed with SPSS Statistics 26. Data is given as mean or median with range from the lowest to the highest value. Statistical significance was defined as *p* < 0.05. To investigate risk factors for vasospasm associated infarction after IAN treatment binary logistic regression was used. Variables that were analysed were age, smoking, Fisher grade,^7^ WFNS World Federation of Neurosurgical Societies, anterior/posterior circulation, spasm debut and days between spasm debut and IAN.

### Ethical considerations

This study was approved by the Ethics Board of the University of Gothenburg, The Sahlgrenska Academy, Gothenburg, Sweden (Dnr 625-16 and 455-12).

## Results

### Patients and demographics

During the 11-year study period, 48 patients with IAN treatment for severe CVS were detected, see Table 1 for the patients' clinical characteristics.

**Table 1.** Clinical characteristics of the patients treated with intra-arterial nimodipine (IAN) at our institution.

	IAN (*n*=48)
Age, mean±SD, (range)	53.7±7.8 (24–80)
Gender, *n* (%)	
Female	34 (71%)
Male	14 (29%)
Hypertension, *n* (%)	17 (35%)
Smoking, *n* (%)	17 (35%)
WFNS, *n* (%)	
1	17 (35%)
2	7 (15%)
3	4 (8%)
4	8 (17%)
5	12 (25%)
Fisher, *n* (%)	
1	1 (2%)
2	4 (8%)
3	4 (8%)
4	39 (81%)
Aneurysm	
Anterior circ, *n* (%)	40 (83%)
Posterior circ, *n* (%)	8 (17%)
Aneurysm treatment	
Endovascular coiling	39 (81%)
Microsurgical clipping	9 (19%)

circ: circulation; SD: standard deviation: WFNS World Federation of Neurosurgical Societies.

Hydrocephalus was seen in 33 (69%) patients treated with ventricular drainage, lumbar drainage or shunt whereas five (10%) patients had incipient or mild hydrocephalus with no treatment and there were 10 (21%) patients with no hydrocephalus.

Median time to clinical deterioration (confusion, decreased level of consciousness, hemiparesis, aphasia) in patients who were conscious and could be neurologically examined, was 6 days (range 2–30 days). Eleven patients were sedated and could not be assessed neurologically. Two patients were assessed coming into the hospital in vasospastic phase and five patients had an anamnesis of headache 3 days to 1 month before admission to hospital having a so-called 'warning leak'. Clinical and radiological characteristics can be seen in Table 2.

**Table 2.** The debut of cerebral vasospasm (CVS), the number of procedures per patient, the vasospastic territory and the distribution of intra-arterial nimodipine (IAN).

Vasospasm debut days after SAH on TCD/radiology, median (range)	5 (0–30)
IAN days after SAH, median (range)	7 (3–30)
IAN days after vasospasm debut, median (range)	2 (0–13)
IAN total number of procedures	124
Number of procedures/patients, median (range)	2 (1–10)
1	17 (35%)
2	13 (27%)
3	6 (13%)
4	7 (15%)
5	1 (2%)
6	1 (2%)
7	2 (4%)
10	1 (2%)
Dose/procedure, median (range)	4 mg (2–9)
Vasospastic territory, *n* (%)	
ACA	116 (46%)
ICA	16 (6%)
MCA	98 (39%)
Posterior circulation	24 (9%)
IAN distribution, *n* (%)	
ACA	48 (24%)
ICA	104 (53%)
MCA	29 (15%)
Posterior circulation	16 (8%)

ICA: internal carotid artery; SAH: subarachnoid haemorrhage: TCD: Transcranial doppler, ACA: Anterior cerebral artery, MCA: Middle cerebral artery.

**Figure 2.**
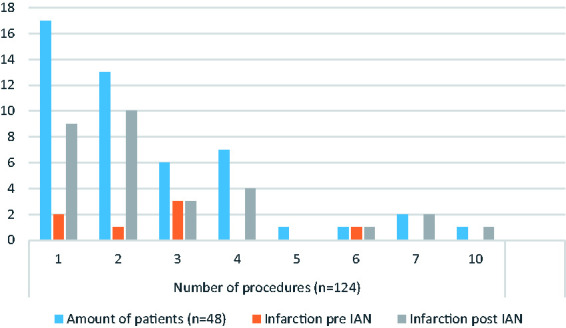
Number of procedures per patient in correlation to infarction both before and after intra-arterial nimodipine (IAN) treatment.

Infarction or ischaemia on CT was seen in 83% of the patients; 21% had infarction before IAN, 27% after IAN and 35% both before and after IAN. Figure 2 describes the relationship between the number of procedures and number of patients developing infarctions both pre- and post-IAN treatment. Infarction after the IAN treatment related to the treated vasospastic vessel was seen in 29 (60%) patients.

The clinical outcomes at both 30 days and 6 months after the procedure are shown in Table 3.

**Table 3.** Clinical outcome in the intra-arterial nimodipine (IAN) group.

	IAN, *n* (%)
mRS 0–2 (30 d)	12 (25%)
mRS 3–5 (30 d)	32 (67%)
mRS 0–2 (6 m)	22 (47%)
mRS 3–5 (6 m)	20 (42%)
Mortality (30 d)	4 (8%)
Mortality (6 m)	6 (13%)

d: days; m: months; mRS: modified Rankin Scale.

Procedure-related complications were seen in 10 (21%) patients, in 10 (8%) procedures respectively, where one patient with vasospasm in ACA acquired an embolus in MCA during the procedure and later developed infarct in the MCA territory. Other complications seen were dissection and pseudoaneurysm at the puncture site of femoral artery, embolus to an intracranial artery during the procedure, in-stent thrombosis in a previously treated aneurysm, thrombosis, occlusion of an artery due to exaggerated vasospasm and in a single patient one of the two coils became loosened during the IAN procedure.

## Discussion

A majority of the patients (60%) in this study developed cerebral infarction associated with vasospasm after the treatment with IAN. A good outcome was seen in 25% (*n* = 12) at 30 days and in 47% (*n* = 22) at 6 months. Four patients (8%) died within 30 days. Procedure-related complications were seen in 10 (21%) patients in 10 (8%) IAN procedures.

Previous studies report variable results regarding vasospasm-related infarction. Hänggi et al.^13^ and Andereggen et al.^19^ report infarctions in 61% of patients, which is in line with our study (60%). Since vasospasm is a major cause of infarction, a high number of infarctions detected is not surprising. It should be noted that even lower number of infarctions after the treatment has been reported, for example both Bashir et al.^1^ and Ditz et al.^17^ report lower figures of infarction: 47% and 48% respectively. Furthermore, Cho et al.^15^ detected infarction only in one-fifth of the patients (Table 4). However, these studies appear to have some different criteria for treatment with IAN, which could have affected the results. In addition, a more complex pathophysiology including other mechanisms than only vasospasm may lead to cerebral ischaemia. Even though the studies in Table 4 showed angiographic improvement in 31 (96%) of patients, the majority still developed infarction. This can be a result of delayed initiation of treatment, irregular repeated treatments and/or no effect of IAN despite satisfactory angiographic results. Furthermore, if the CVS-associated ischaemic damage has already occurred before treatment with IAN, it is no wonder that despite the angiographic improvement a lower response than expected is seen. A CT might be useful in between treatments with IAN, however this is not the best option when it comes to infarctions early on, MRI might be more useful but due to logistical issues this is not as widely used at this institution. Nimodipine tends to have a short-lived effect which is not enough to maintain sufficient cerebral perfusion.^12^ It has been attempted to infuse nimodipine continuously but having a catheter placed in the artery for several days entails a risk of thromboembolism, catheter occlusion and infection.^17^

**Table 4.** Studies with cerebral vasospasm (CVS) after aneurysmal subarachnoid haemorrhage (SAH) and intra-arterial nimodipine (IAN) treatment.

	CG	Pat	AI	CI	Infarct	mRS 0–2/GOS >4	mRS 0–2 (3–10 m)	Mortality in hosp/30 d	CVS onset d after SAH	Complication
Biondi et al., 2004^19^	No	25	43 %	76 %	–	–	3–6 m: 72%	2 (8%)	Mean 7 ± 3 days	No
Hänggi et al., 2008^13^	No	18	77 %	11 %	61 %	61 %	3 m: 61 %	1 (5%)	–	No
Kim et al., 2009^14^	No	19	79 %	68 %	–	79 %	–	0 (0%)	Mean 9.6 ± 3.1	No
Cho et al., 2011^15^	No	42	82 %	68 %	21 %	76 %	6 m: 85 %	1 (2%)		3 (3% of procedures)
Bashir et al., 2016^1^	No	25	96 %	12 %	48 %	4 %	3 m: 20%	9 (36%)	Median 8	No
Andereggen et al., 2017^20^	Yes, 52	31	31 %	–	61 %	25 %	10 m: 60%	16 (20%)	–	5 (16% of pat)
Ditz et al., 2018^17^	Yes, 15	15	93 %	–	47 %	0 %	3/6 m: 12%/36%	1 (7%)	Median 6 (0–10)	1 (1.5% of procedures)
Our study, 2020	Yes, 7	48	–	–	60 %	25 %	6 m: 47%	2 (4%)/4 (8%)	Median 5 (0–30)	10 (21% of pat, 8% of procedures

AI: angiographic improvement; CG: control group; CI: clinical improvement; CVS: cerebral vasospasm; d: days; GOS: Glasgow Outcome Scale; hosp: hospital; infarct: infarct related to vasospasm; m: months; mRS: modified Rankin Scale; pat: number of patients.

Symbol - indicates no information.

The number of good outcomes may be affected by a series of other factors such as patient factors and previous medical conditions, severity of initial haemorrhage and different institutional factors like treatment protocols and treatment availability. Bashir et al.^1^ reported a trend toward poorer outcome with increasing time delay from symptomatic CVS to IAN treatment. In this study treatment with IAN was started at a median of 2 days after signs of vasospasm on TCD or radiology. This means that IAN at our centre is initiated late which can have an effect on the high number of infarctions and poor outcomes. However, this time range is difficult to determine precisely retrospectively because the time for treatment is not always clearly documented and the criteria for treatment is not standardised. These patients treated with IAN are often relatively young and already in a devastating state regarding refractory vasospasm to treatment and upcoming ischaemia, which is why even desperate attempts with IAN are sometimes carried out. This can be seen as repeated treatments of up to 10 procedures in the present study. However, the number of good outcomes could be even lower without the treatment.

Complications in this study were seen in 10 (21%) patients in 10 (8%) IAN procedures respectively. Severe complications were rare. Previous studies report low rates of procedure-related complications which is why IAN is considered as a safe and feasible treatment. Complications that are included in studies and reporting of complications may differ between studies. This could partly explain the somewhat higher number of complications in our study compared with the studies in Table 4. The complications reported in some of the studies in Table 4 are similar to ours: occlusion with subsequent infarction, thrombosis, dissection in the access vessel and pseudoaneurysm at the puncture site of femoral artery. A low mortality rate was seen both in the present study and in the literature, which supports the nature of IAN as a safe treatment.

We did not find any statistically significant risk factors in variables we analysed for developing infarction after treatment with IAN unlike some previous studies. For example, Andereggen et al.^19^ identified severe SAH (Hunt-Hess scale ≥3) (54% of the patients), number of treatments (mean 2.5±1.7), number of vessels treated and number of vascular territories treated as risk factors associated with infarction. The statistical relation can be explained by the fact that the severity of vasospasm has an effect on developing infarction and, furthermore, the grade of vasospasm and distribution in several vessels may have an effect on the number of treatments and number of vessels treated. One patient in our study went through five IAN treatments; this was the only patient that did not develop an infarction radiologically. In all other patients, we could conclude that almost half or more developed an infarction in the territory where they were previously treated. Similar numbers to Andereggen et al.^19^ were seen in our study where severe SAH as high WFNS (grade 4–5) was seen in 42% of the patients and mean number of treatments 2.6±1.9, although these were not statistically significant. Risk factors for only cerebral vasospasm include severe SAH on CT (Fisher scale 3), cigarette smoking, hypertension and left ventricular hypertrophy.^7^ In the present study severe SAH on CT was seen in 90% of the patients, current or history of smoking in 50% of the patients and hypertension in 35% of the patients.

IAN has been tested and adopted in clinical practice despite a lack of randomised trials and strong evidence. Biondi et al.^18^ were the first to describe effective treatment results of IAN therapy in 2004. Being a relatively new treatment method with no strong evidence it is clear that more studies with larger patient groups are needed regarding effectiveness of the treatment. Other aspects such as time-delay from symptom debut to IAN and the effect duration of IAN would also be of interest. Furthermore, the question remains regarding when intervention should be initiated; clinical deterioration is the most important sign, assuming that other factors are ruled out. However, detecting clinical signs in unconscious patients and patients with already neurological signs is difficult. Moreover, TCD can be unreliable and CTA may not only overestimate but also underestimate the severity of vasospasm, whereas CTP can provide good information on relatively large perfusion defects but not on smaller ones. Furthermore, how aggressive should the approach to treatment be; in our departments of neurosurgery and interventional radiology centre nimodipine is tried first and if no effect, PTA can be considered. Some other centres perform PTA directly and studies with early PTA have shown good results with a lower rate of DCI and direct reversal of vasospasm with durable result and less retreatment.^20^,^21^ However, there is a possibility of risk for severe consequences with the procedure. Finally, little data about repeated treatments exist and the question about how many injections and how often IAN should be performed still remains. Given that there is no consensus of standardised treatment protocols for patients with cerebral vasospasm in and between centres, the comparability and validity of study results are limited.

### Strengths and limitations

Considering strengths, first, compared to published data with similar studies to date, we have the largest patient population regarding patients treated with IAN. Second, none of the patients were lost during the follow-up period. Finally, since all patients were treated at the same centre with specific indication for treatment, the selection of patients is consecutive.

Considering limitations, first, since it is a single-centre retrospective study the number of patients (*n* = 48) may limit generalisability. Being a retrospective study there is a risk of selection bias and lack of information in the medical records. Use of past written medical records can leave room for subjective interpretation as we had to rely on others’ accurate recordkeeping; this means that there is a risk of different outcome numbers. Finally, the follow-up time at 6 months is not exactly the same for all patients due to the fact that not all patients were at a medical institution at that exact time point and therefore no medical records were available.

## Conclusion

In the present study, infarction after the IAN treatment was seen in a majority (60%) of the patients. Procedure-related complications were seen in 10 patients (21%) in 10 procedures (8%). A good clinical outcome was seen in almost half of the patients later on. This retrospective study has some important limitations and further randomised controlled trials are needed to evaluate the effectiveness of the treatment and to investigate more effective treatment strategies to improve patient outcomes.
